# Coupling photosynthetic physiology and C_4_ enzyme regulation enhances grain yield in no-tillage intercropped maize in an irrigated oasis region

**DOI:** 10.3389/fpls.2026.1780528

**Published:** 2026-04-24

**Authors:** Congcong Guo, Yan Wang, Xiaoyuan Bao, Hong Fan, Yali Sun, Wei He, Chunsheng Bai, Fuyang Cui, Chengxin Bai, Xinying Li, Cai Zhao

**Affiliations:** 1 State Key Laboratory of Aridland Crop Science, Gansu Agricultural University, Lanzhou, China; 2 Seed Industry Research Institute of Gansu Provincial University, Lanzhou, China

**Keywords:** C_4_ enzymes, intercropped maize, irrigation, no-tillage, photosynthetic physiology, yield

## Abstract

**Introduction:**

The Hexi Oasis irrigation area is endowed with abundant light and heat resources, making it suitable for intercropped maize systems. However, increasing water scarcity and the need for irrigation reduction have made the conventional full-irrigation, high-yield pathway difficult to sustain. Under reduced irrigation, yield penalties may occur because water limitation directly constrains stomatal opening and carbon assimilation during the critical silking–grain filling period. Whether intercropped maize can stabilize yield under limited water supply through coordinated maintenance of photosynthetic performance and regulation of key C_4_ enzymes remains unclear.

**Methods:**

To address this gap, a field experiment was conducted in 2022–2024 to systematically evaluate the coupled effects of tillage (no-tillage, NT; conventional tillage, CT), cropping pattern (intercropping, IM; monocropping, SM), and three irrigation regimes (low, I1; medium, I2; high, I3) on maize grain yield, photosynthetic physiology, and key enzyme regulation.

**Results:**

The results showed that compared with the NT×IM×I2 group, the grain yields of the CT×IM×I2 and NT×SM×I2 groups increased significantly by 10.5% and 27.2% respectively. During the silking–grain filling stage, this treatment maintained the highest relative chlorophyll content, net photosynthetic rate, stomatal conductance and effective quantum yield of PSII photochemistry, along with the lowest intercellular CO_2_ concentration and quantum yield of non-regulated energy dissipation. Enzyme activities of phosphoenolpyruvate carboxylase, ribulose-1,5-bisphosphate carboxylase/oxygenase, and pyruvate phosphate dikinase increased by 6–11%, 8–10%, and 9–14%, respectively, with corresponding gene expression upregulated by 30–80%.

**Conclusion:**

In summary, NT combined with moderate irrigation enhanced intercropped maize yield stability under limited water supply through a dual mechanism of “photosynthetic performance maintenance + C_4_ enzyme activity/transcription enhancement.”

## 1 Introduction

The contradiction between the rapidly growing population and the shrinking arable land area poses a serious threat to food security. Maximizing crop yields through the efficient use of limited resources in agricultural production practices is a crucial pathway to achieving sustainable agriculture and safeguarding national food security (Rudel et al., 2009, pp. 1970–2005). Intercropping systems can optimize the utilization of light, heat, water, and nutrients by exploiting niche separation mechanisms, thereby allowing two crops to complement each other spatially and temporally (Raza et al., 2022). In the Hexi Oasis irrigation region, where light and heat resources are abundant, intercropping maize is particularly suitable. However, the region faces severe water scarcity (Pan et al., 2024), which restricts the sustainable development of the maize industry. Therefore, investigating intercropping techniques for stable maize production under limited irrigation and clarifying the theoretical basis of water-saving efficiency has become an urgent issue to address.

Intercropping and no-tillage are widely recognized as key practices for water saving and yield stability because they improve resource-use efficiency and conserve soil moisture. Intercropping enhances complementary use of water and nutrients through differences in root architecture and canopy structure, which can sustain post-flowering source strength and favor assimilate allocation to grains (Chen et al., 2024; Khashi U Rahman et al., 2025). No-tillage can slow chlorophyll decline, maintain photosynthetic duration in mid-to-late growth stages, and support assimilate translocation, thereby benefiting yield formation (Li et al., 2022). Irrigation level further regulates these processes: moderate deficit irrigation may promote deeper rooting and maintain leaf function, whereas stronger water limitation can constrain stomatal conductance and carbon assimilation, despite reports that mild water stress may transiently stimulate activities of key photosynthetic enzymes (Ghannoum, 2009; Faralli et al., 2019; Qu et al., 2020; Guo et al., 2024b, 2024a). However, most evidence has been derived from monocropped maize under conventional tillage or full irrigation, and systematic field-based evidence for the combined effects of the no-tillage × intercropping × limited irrigation triad remains scarce. More importantly, whether this agronomic combination can coordinately regulate photosynthesis at both physiological and molecular levels to compensate for potential biomass loss under reduced irrigation is still unclear.

Photosynthetic performance represents the primary physiological bottleneck determining maize yield formation. More than 60% of dry matter accumulation during the grain-filling stage originates from post-anthesis photosynthesis, which coincides with the water-sensitive period in the Hexi irrigation region (Jiao et al., 2024). Under limited irrigation, no-tillage can effectively improve the photosynthetic performance of monocropped maize by regulating soil physicochemical properties and enhancing soil water and nutrient availability, thereby sustaining relatively high grain yields (Adil et al., 2024). As a C4 crop, maize exhibits photosynthetic efficiency that is highly dependent on the activity and gene expression of PEPC, pyruvate phosphate dikinase (PPDK), and Rubisco (Wang et al., 2011). Meanwhile, chlorophyll fluorescence parameters can dynamically capture changes in PSII photochemical efficiency and photoprotective capacity (Murchie and Lawson, 2013). Theoretically, the combined effects of no-tillage improving rhizosphere water status, intercropping optimizing canopy light distribution, and moderate irrigation inducing mild reversible stress may jointly activate a cascade pathway of “upregulated C4 enzyme gene expression → enhanced enzyme activity → increased net photosynthetic rate (Pn) and effective quantum yield of PSII photochemistry (Y(II)),” thus achieving photosynthetic compensation under limited water supply (Cacefo et al., 2019). However, such mechanisms remain to be validated through field experiments.

However, the region faces severe resource-based water scarcity, which constrains the sustainable development of the maize industry. Therefore, under limited irrigation, identifying agronomic optimizations that enhance photosynthetic efficiency in intercropped maize to ensure yield stability is a key challenge for advancing water-saving, high-efficiency agriculture in this area. We hypothesize that the no-tillage–intercropping system under moderate irrigation maintains leaf photosynthetic performance through a photosynthetic compensation mechanism mediated by coordinated upregulation of key C_4_ enzyme activity and gene expression, thereby stabilizing grain yield under limited water supply. To this end, we systematically investigated: (1) how the coupling of no-tillage and intercropping regulates maize gas-exchange, chlorophyll fluorescence, and relative chlorophyll content (SPAD value) dynamics under limited irrigation; (2) whether these physiological responses are coordinated with changes in the activities of PEPC, PPDK, and Rubisco and the transcription of their corresponding genes (pepc, ppdk, rbcL); and (3) the relative contributions and path coefficients linking the photosynthesis–enzyme activity–molecular regulation network to grain yield and its components. The results aim to elucidate the photosynthetic physiological and molecular mechanisms by which the “no-tillage + moderate irrigation + intercropping” system achieves water-saving yield stability, providing quantifiable theoretical thresholds and practical pathways for maize production in the arid Northwest.

## 2 Materials and methods

### 2.1 Plant materials

The maize cultivar used in the experiment was Xianyu 335 (*Zea mays* L.), and the pea cultivar was Longwan No. 1 (*Pisum sativum* L.). Seeds of maize (Xianyu 335) and pea (Longwan 1) were purchased from local commercial suppliers in Lanzhou and Wuwei, Gansu, China. Xianyu 335 is a nationally approved hybrid in China (approval Nos. Guoshenyu 2004017 and Guoshenyu 2006026), and Longwan No. 1 is nationally registered as a non-major crop variety (registration No. GPD-Pea(2018)620005). No wild materials were collected and no permits were required.

### 2.2 Experimental design

The experiment was conducted in 2022–2024 at the “Oasis Agricultural Research and Teaching Base of Gansu Agricultural University” in Huangyang Town, Liangzhou District, Wuwei City, Gansu Province, China (37°30′N, 103°5′E). The site is located in a cold temperate arid climate zone, with an average annual precipitation of approximately 156 mm, annual evaporation of about 2400 mm, total sunshine duration of 2969.2 h, mean annual temperature of 7.2 °C, and a frost-free period of 156 days. The region has abundant solar radiation, making it well suited for maize intercropping systems. Local agricultural production is predominantly based on conventional tillage with plowing, and plastic film mulching is commonly applied.

The soil type at the experimental site was irrigated desert soil. In 2024, the nutrient contents of the plow layer were as follows: total nitrogen, 0.89 g kg^-^¹; available phosphorus, 24.98 mg kg^-^¹; available potassium, 138.44 mg kg^-^¹; organic matter, 14.53 g kg^-^¹; and bulk density, 1.24 g cm^-^³. Variations in temperature and precipitation at the site throughout the 2024 growing season are shown in **Supplementary Figure S1**. Plastic film mulching was applied to all treatments following the local standard practice. Film specifications (material and thickness), mulching timing, and field management were kept consistent across treatments; thus, mulching was considered a uniform background practice rather than an experimental factor.

Based on a long-term experiment initiated in 2015, this study adopted a split-split-plot design with three factors. The main plots included two tillage practices: no-tillage (NT, where maize was directly sown the following year onto the residual film after the previous maize harvest without tillage) and conventional tillage (CT, where the field was deep-plowed after the previous maize harvest, and new plastic film was applied after land preparation before sowing the following year). The subplots consisted of two planting patterns: intercropped maize (IM) and monocropped maize (SM). The sub-subplots were assigned three irrigation levels: low irrigation (I1, 4500 m^3^ ha^-1^), moderate irrigation (I2, 4950 m^3^ ha^-1^), and high irrigation (I3, 5400 m^3^ ha^-1^), with the high irrigation treatment representing the local conventional irrigation level. The three irrigation levels were defined around the local conventional irrigation quota (I3) to represent feasible irrigation-reduction scenarios: I2 and I1 correspond to 8.3% and 16.7% reductions relative to I3, respectively, and were therefore termed “moderate” and “low” irrigation. In total, 12 treatment combinations were established, each replicated three times, resulting in 36 plots. Each plot had an area of 63 m² (7 m × 9 m). The treatment codes are provided in **Supplementary Table S1**.

The planting density of monocropped maize was 90,000 plants ha^-^¹, with a row spacing of 40 cm and a plant spacing of 27 cm. In the maize–pea intercropping system, a 3:4 planting ratio (three rows of maize to four rows of pea) was adopted, with a spacing of 25 cm between maize and pea rows. The planting density of intercropped maize was 52,000 plants ha^-^¹, while that of pea was 760,000 plants ha^-^¹, with an inter-row ratio of 8:11 between pea and maize strips. The corn was sown on April 18, 2022, April 15, 2023, and April 18, 2024 respectively, and was harvested on September 24, 2022, September 22, 2023, and September 27, 2024 respectively. After harvest, all crop residues were removed from the field. Within each block, main plots were randomly allocated to tillage treatments, and subplots and sub-subplots were randomly assigned within each main plot. Each sub-subplot measured 6.0 m × 5.0 m, with a row spacing of 0.60 m and an in-row plant spacing of 0.25 m. Two outer rows on each side of the plot and 0.5 m at both ends were treated as border rows and excluded from sampling. All statistical analyses were performed on plot-level means, with plant-level measurements averaged within each plot prior to analysis, so that the experimental plot served as the statistical unit.

All irrigation treatments were applied using drip irrigation under plastic film, with water volume monitored by flow meters. Specific irrigation quotas at each growth stage are provided in **Supplementary Table S2**. The maize fertilization regime followed local conventional management, with a total nitrogen application of 360 kg ha^-^¹, split at a ratio of 3:5:2 across the V6 (early vegetative) and VT (tasseling) stages, and the grain-filling stage. Phosphorus fertilizer was applied at an N:P ratio of 2:1, equivalent to 180 kg ha^-^¹, incorporated entirely as a basal application. For pea, total nitrogen and phosphorus application rates were 90 kg ha^-^¹ and 45 kg ha^-^¹, respectively, both applied entirely as basal fertilizer across all treatments. The chemical nitrogen fertilizers applied included urea and diammonium phosphate. White agricultural plastic film was used for mulching, with a width of 120 cm and a thickness of 0.01 mm. Given the high native potassium content of the soil in this region, no potassium fertilizer was applied. Pest, disease, and weed management practices were carried out according to local conventional tillage practices.

### 2.3 The yield and yield components

At maturity, a 5 m row length in the center of each plot (one film width of 1.4 m, totaling 7 m²) with continuous plants and no gaps was selected as the sampling area for yield measurement. Ears were harvested manually, shelled on site, and cleaned of impurities. Fresh grain weight was measured using an electronic balance with an accuracy of 0.01 kg. A 500 g grain subsample was randomly collected, inactivated at 105 °C for 30 min, and then oven-dried at 80 °C to a constant weight to determine actual moisture content. Grain weight was converted to a standard moisture content of 14% according to national maize yield determination standards and further converted to yield per hectare (kg ha^-^¹).

### 2.4 Leaves relative chlorophyll content

At the jointing, big-tassel, silking, grain-filling, and dough stages, the relative chlorophyll content (SPAD value) of maize leaves was measured on sunny mornings between 9:30 and 11:30. SPAD values were determined using a SPAD-502 Plus chlorophyll meter. For each measurement, three representative maize plants of uniform growth were randomly selected, and three readings were taken per plant; the average value was recorded as the observation, avoiding interference from leaf veins. At the jointing stage, measurements were taken on the second fully expanded leaf from the top of the plant. At the big-tassel, silking, grain-filling, and dough stages, measurements were taken on the ear leaf, with the reading location at the middle portion of the leaf, avoiding veins and leaf margins.

### 2.5 Leaves gas exchange parameters

At the jointing, big-tassel, silking, grain-filling, and dough stages, the net photosynthetic rate (Pn), transpiration rate (Tr), intercellular CO_2_ concentration (Ci), and stomatal conductance (Gs) of maize leaves were measured between 9:30 and 11:30 a.m. on sunny days using a LI–6800XT (LI-COR Biosciences, Lincoln, NE, USA) portable photosynthesis system. Three representative maize plants of uniform growth were randomly selected for measurement, with care taken to avoid leaf veins. During measurement, the leaf chamber area was set to 2 cm², with an airflow rate of 500 μmol s^-^¹. Leaf temperature was maintained equal to air temperature, and relative humidity was controlled at 55%. The leaf chamber photosynthetic photon flux density (PPFD) was set to 1,500 μmol m^-^² s^-^¹ using the built-in red–blue LED light source. The reference CO_2_ concentration was maintained at 400 μmol mol^-^¹, with a flow rate of 500 μmol s^-^¹ and leaf temperature controlled at 25 ± 1 °C. Relative humidity in the chamber was kept between 55% and 65%. Leaves were allowed to equilibrate in the chamber until CO_2_ and H_2_O exchange signals were stable for at least 2–3 min before logging data. At the jointing stage, the second fully expanded leaf from the top was selected, while at the big-tassel, silking, grain-filling, and dough stages, the ear leaf was measured at the middle portion, avoiding veins and leaf margins.

At the jointing, big-tassel, silking, grain-filling, and dough stages, the net photosynthetic rate (Pn), transpiration rate (Tr), intercellular CO_2_ concentration (Ci), and stomatal conductance (Gs) of maize leaves were measured between 9:30 and 11:30 a.m. on sunny days using a LI–6800XT portable photosynthesis system (LI-COR Biosciences, Lincoln, NE, USA). Three representative maize plants with uniform growth were randomly selected for measurement, and the middle portion of the target leaf was clamped while avoiding major veins and leaf margins. The leaf chamber area was set to 2 cm^2^ with a flow rate of 500 μmol s^-1^. The chamber photosynthetic photon flux density (PPFD) was set to 1500 μmol m^-2^ s^-1^ using the built-in red–blue LED light source, and the reference CO_2_ concentration was maintained at 400 μmol mol^-1^. During measurements, the chamber temperature was controlled at 25 ± 1 °C and relative humidity was maintained at 55–65%. Leaves were allowed to equilibrate until CO_2_ and H_2_O exchange signals were stable for at least 2–3 min before data logging. At the jointing stage, the second fully expanded leaf from the top was measured, whereas at the big-tassel, silking, grain-filling, and dough stages, the ear leaf was measured.

### 2.6 Leaves chlorophyll fluorescence parameters

At the jointing, big-tassel, silking, grain-filling, and dough stages, chlorophyll fluorescence parameters of maize leaves were measured between 9:30 and 11:30 a.m. on sunny days using an FMS-II pulse-modulated fluorometer (PAM-2500). Three representative maize plants of uniform growth were randomly selected, and each was measured three times; the average value was recorded as the observation, avoiding interference from leaf veins. At the jointing stage, the second fully expanded leaf from the top was selected, while at the big-tassel, silking, grain-filling, and dough stages, the ear leaf was measured at the middle portion of the leaf, avoiding veins and margins.

Prior to measurement, target leaves were dark-adapted for 30 min using dark adaptation clips. Thereafter, a measuring light (<0.05 μmol m^-^² s^-^¹) followed by a saturating pulse light (6000 μmol m^-^² s^-^¹) was applied to obtain the minimum fluorescence (Fo) and maximum fluorescence (Fm) under dark-adapted conditions. The maximum photochemical efficiency was then calculated as: Fv/Fm = (Fm – Fo)/Fm

Subsequently, actinic light (1000 μmol m^-^² s^-^¹) was applied for 3 min. Once the fluorescence signal stabilized, the steady-state fluorescence (Fs) was recorded. A saturating pulse light (6000 μmol m^-^² s^-^¹) was then applied to determine the maximum fluorescence under light (Fm′) and the minimum fluorescence under light (Fo′). The following parameters were calculated:

Actual photochemical efficiency of PSII: Y(II)=(Fm′-Fs)/Fm′

Quantum yield of non-regulated energy dissipation: Y(NO)= Fs/Fm

Quantum yield of regulated energy dissipation: Y(NPQ)=1 − Y(II) − Y(NO)

Relative electron transport rate: ETR=PAR×Y(II)×0.84×0.5

where PAR represents photosynthetically active radiation (1000 μmol m^-^² s^-^¹), 0.84 is the leaf absorption coefficient, and 0.5 assumes equal partitioning of excitation energy between PSI and PSII.

### 2.7 The activity of key photosynthetic enzymes

At the jointing stage of maize, the second fully expanded leaf from the top was sampled, while at the silking and grain-filling stages, fresh samples were collected from the middle section of ear leaves for the determination of key photosynthetic enzymes. Three replicates were taken for each treatment, with 0.2 g of tissue per replicate. Samples were immediately frozen in liquid nitrogen and then stored in an ultra-low temperature freezer at –80°C to preserve tissue structure and biological activity.

The activities of PEPC, PPDK, and Rubisco were measured using assay kits supplied by Suzhou Comin Biotechnology Co., Ltd., following the manufacturer’s instructions.

### 2.8 qRT-PCR

To analyze the expression dynamics of key photosynthetic enzyme genes in maize leaves under different tillage practices, planting patterns, and irrigation regimes, samples were collected at three growth stages: jointing, silking, and grain filling. For each treatment, three maize plants of uniform growth were randomly selected. At the jointing stage, the second fully expanded leaf from the top was sampled; at the silking and grain-filling stages, the middle portion of the ear leaf was collected. After removing the veins, 0.2 g of fresh tissue was quickly weighed, flash-frozen in liquid nitrogen, and stored at –80 °C. Total RNA was extracted from maize leaves using the HiScript^®^ II Q RT SuperMix for qPCR (+gDNA wiper) kit (Nanjing). RNA quality was assessed with an Agilent 2100 Bioanalyzer and verified by RNase-free agarose gel electrophoresis. RNA was then reverse-transcribed into cDNA using the FastPure^®^ Plant Total RNA Isolation Kit (Nanjing), following the instructions provided with the PrimeScript reverse transcription kit. The cDNA synthesis reaction system (20 µL) contained 4 µL of 5× HiScript II qRT SuperMix II, 4 µL of 4× gDNA Wiper Mix, 12 µL of RNase-free H_2_O, and RNA (1000 ng). Reaction conditions were 50 °C for 15 min and 85 °C for 5 s. The cDNA products were stored at –20 °C until use.

Subsequently, quantitative real-time PCR (qRT-PCR) was performed. The maize coding sequences were obtained from the MaizeGDB database (http://www.maizegdb.org/), and primers were designed using Primer Premier 5.0 (Canada) and synthesized by Shanghai OE Biotech Co., Ltd. (primer sequences are provided in **Supplementary Table S3**). The reactions were carried out using the Taq Pro Universal SYBR qPCR Master Mix kit on a LightCycler^®^ 480 II real-time PCR system (Switzerland). The reaction mixture (20 µL) consisted of 10 µL of 2× Taq Pro Universal SYBR qPCR Master Mix, 0.4 µL of 10 µM Primer 1, 0.4 µL of 10 µM Primer 2, 1 µL of cDNA, and 8.5 µL of nuclease-free H_2_O. The PCR program was set as follows: 95 °C for 30 s; followed by 40 cycles of 95 °C for 5 s and 60 °C for 30 s. Gene expression levels were calculated using the 2^-^(ΔΔCt) method, with ZmActin serving as the internal reference gene. Each treatment included three biological replicates. The specific primer sequences are listed in **Supplementary Table S3**.

### 2.9 Statistical analysis

Data were recorded and organized using Microsoft Excel 2019. Duncan’s multiple range tests were performed in SPSS 21.0. Figures and correlation analyses were generated with Origin 2019b and GraphPad Prism 9.0. Results are expressed as mean ± standard error (mean ± SE). One-way ANOVA followed by the least significant difference (LSD) test was used to determine significance at the 95% or 99% confidence level. Image assembly was conducted in Adobe Illustrator 2020.

## 3 Results

### 3.1 Effects of moderate irrigation and no-tillage on grain yield and yield components of intercropped maize

**Figure 1:**
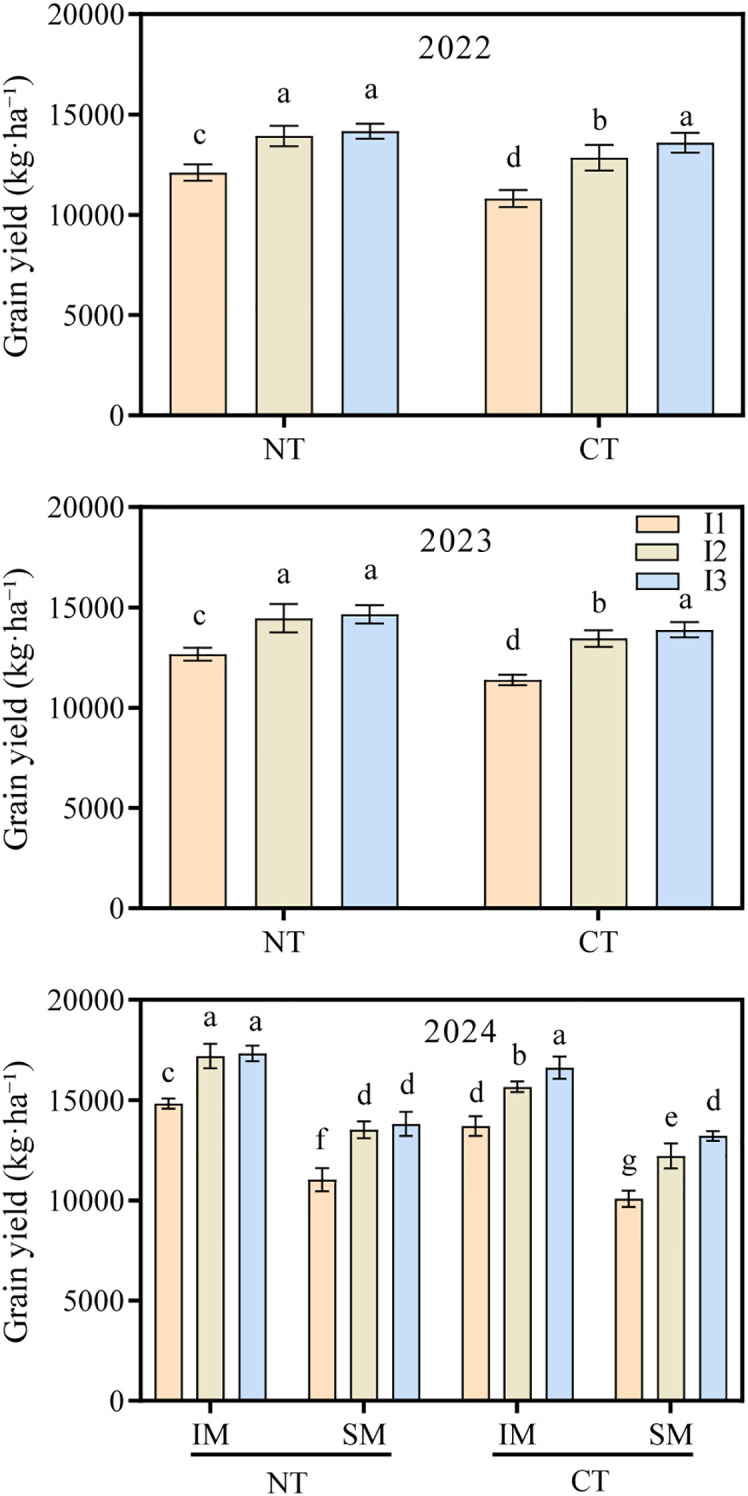
Effect of moderate irrigation under no-tillage on grain yield of intercropped maize in 2022–2024. Differences between treatments are denoted by different lowercase letters, with a significance level of 0.05, and the same applies hereafter.

Moderate irrigation combined with no-tillage significantly affected grain yield in intercropped maize, showing significant two-way but non-significant three-way interactions (**Figure 1**). Data from 2022 indicated that among different farming methods, NT increased grain yield by 7.8% compared with CT. At the same land area, IM produced 29.5% higher yield than SM. Across irrigation regimes, grain yield declined with reduced irrigation: I1 produced 18.9% less yield than I3, whereas I2 did not differ from I3. Within the intercropped system, NT×IM×I2 achieved the highest grain yield, exceeding CT×IM×I2 and NT×SM×I2 by 10.5% and 27.2%, respectively (**Figure 1A**). Similar trends were observed in 2023 and 2024 (**Figures 1B,C**).

The yield advantages of NT and IM were mainly attributable to increases in ear number and 1000-grain weight (**Table 1**). Compared with CT, NT increased ear number and grain weight by 6.0% and 5.8%, respectively; IM further increased these components by 5.8% and 6.2% compared with SM. In contrast, tillage and planting patterns had no significant effect on kernel number per ear. Irrigation exerted a strong influence on yield components: relative to I3, I1 reduced both ear number and grain weight, whereas I2 maintained values similar to I3. Consistently, within the intercropped system, NT×IM×I2 significantly increased ear number (+8.0%) and grain weight (+6.3%) compared with CT×IM×I2.

**Table 1:** Three factors of maize yield under different treatments.

Tillage initiatives	Planting pattern	Irrigation amounts	Ear numbers (ear m^-2^)	Kernel number per spilk	1000-kernel weight (g)
NT	IM	I1	8.1 ± 0.12 d	562.0 ± 18.6 c	436.0 ± 7.5 d
		I2	9.5 ± 0.21 a	643.5 ± 21.2 ab	489.1 ± 18.6 a
		I3	9.6 ± 0.06 a	652.2 ± 17.0 a	494.6 ± 5.0 a
	SM	I1	7.4 ± 0.15 e	528.3 ± 6.9 d	410.7 ± 13.2 f
		I2	9.0 ± 0.10 bc	586.9 ± 14.9 c	461.9 ± 16.5 bc
		I3	9.1 ± 0.20 b	619.7 ± 19.3 b	463.0 ± 21.6 b
CT	IM	I1	7.5 ± 0.15 e	523.5 ± 24.4 d	413.9 ± 8.6 ef
		I2	8.8 ± 0.12 c	620.8 ± 33.0 b	460.2 ± 12.7 bc
		I3	9.1 ± 0.17 b	631.5 ± 21.6 ab	465.5 ± 18.8 b
	SM	I1	7.0 ± 0.10 f	492.1 ± 20.7 e	388.5 ± 7.0 g
		I2	8.3 ± 0.26 d	583.4 ± 16.4 c	343.4 ± 13.5 de
		I3	9.0 ± 0.10 bc	617.9 ± 9.0 b	440.6 ± 10.0 cd
Variance analysis					
Tillage initiatives (T)			**	NS	**
Planting pattern (P)			**	NS	*
Irrigation amounts (I)			**	*	*
T×P			*	NS	**
T×I			*	NS	*
P×I				NS	*
T×P×I			NS	NS	NS

Differences between treatments are denoted by different lowercase letters, with ** and * indicating significant effects of the experimental factors on the parameter at the 0.01 and 0.05 significance levels, respectively, while NS denotes no significant effect of the experimental factors on the parameter. Abbreviations are listed in the Abbreviations section.

### 3.2 Effects of moderate irrigation and no-tillage on photosynthesis and chlorophyll fluorescence of intercropped maize

**Figure 2:**
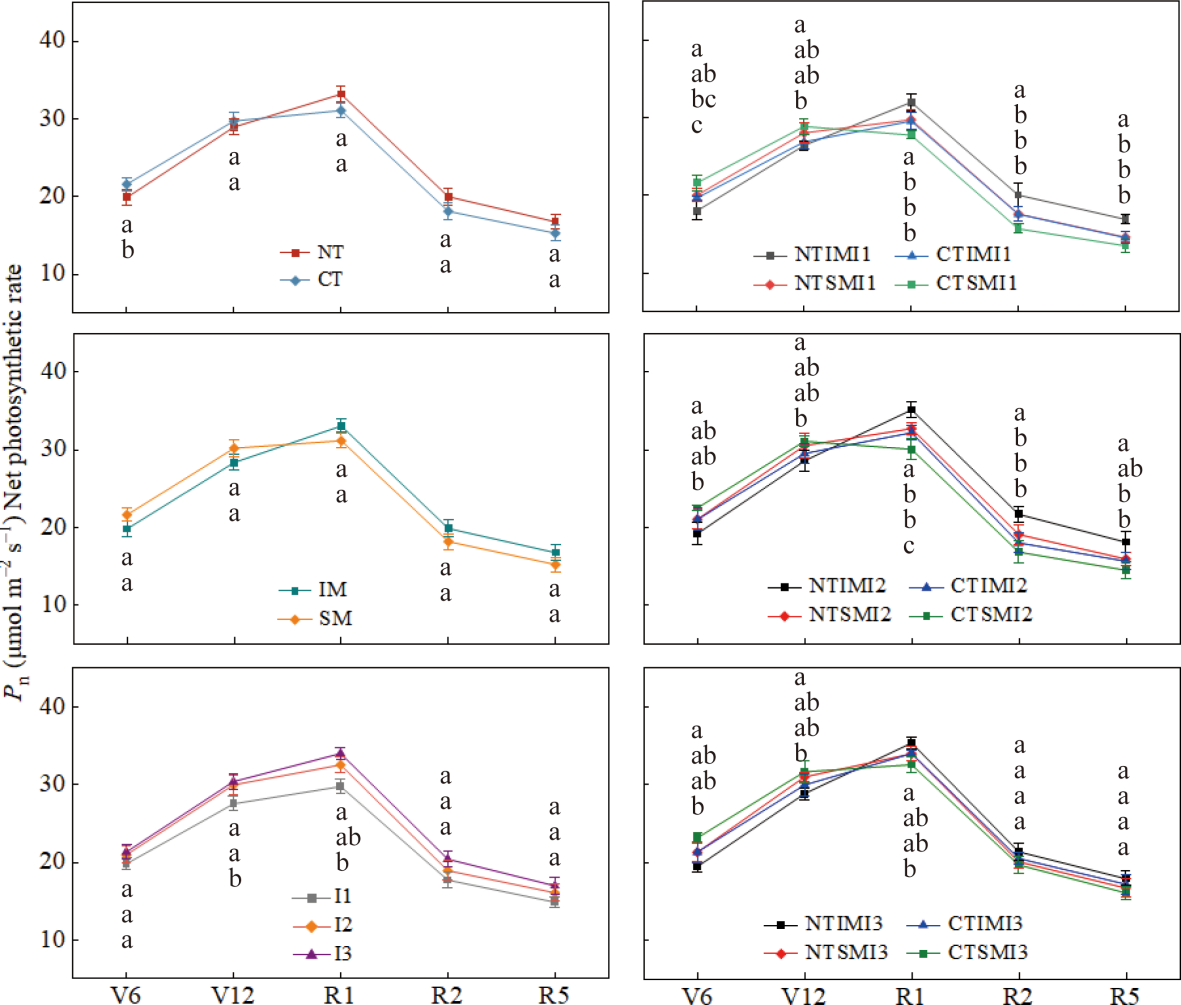
Effect of moderate irrigation under no-tillage on the net photosynthetic rate (Pn) of intercropped maize at different growth stages.

**Figure 3:**
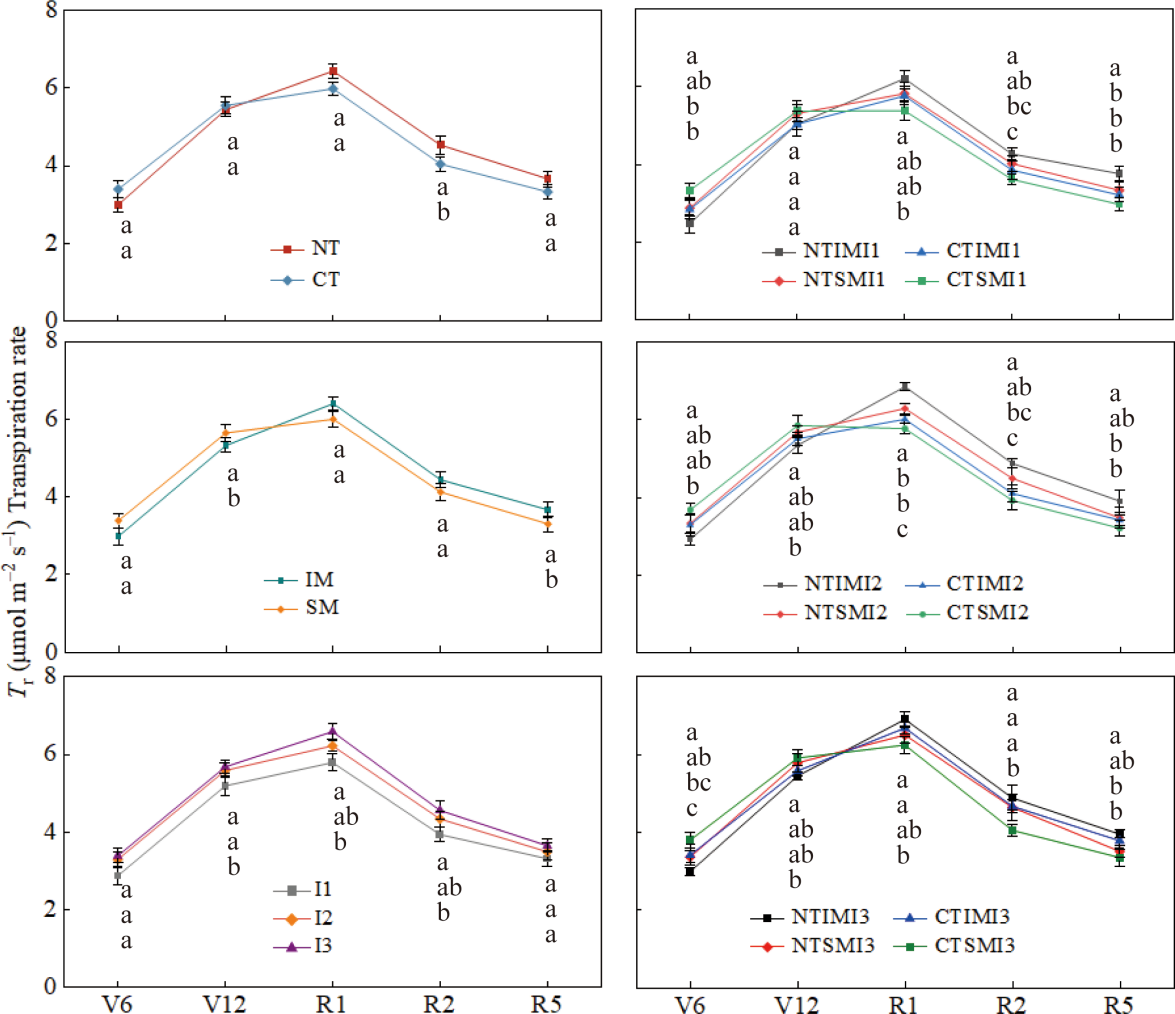
Effect of moderate irrigation under no-tillage on the transpiration rate (Tr) of intercropped maize at different growth stages.

**Figure 4:**
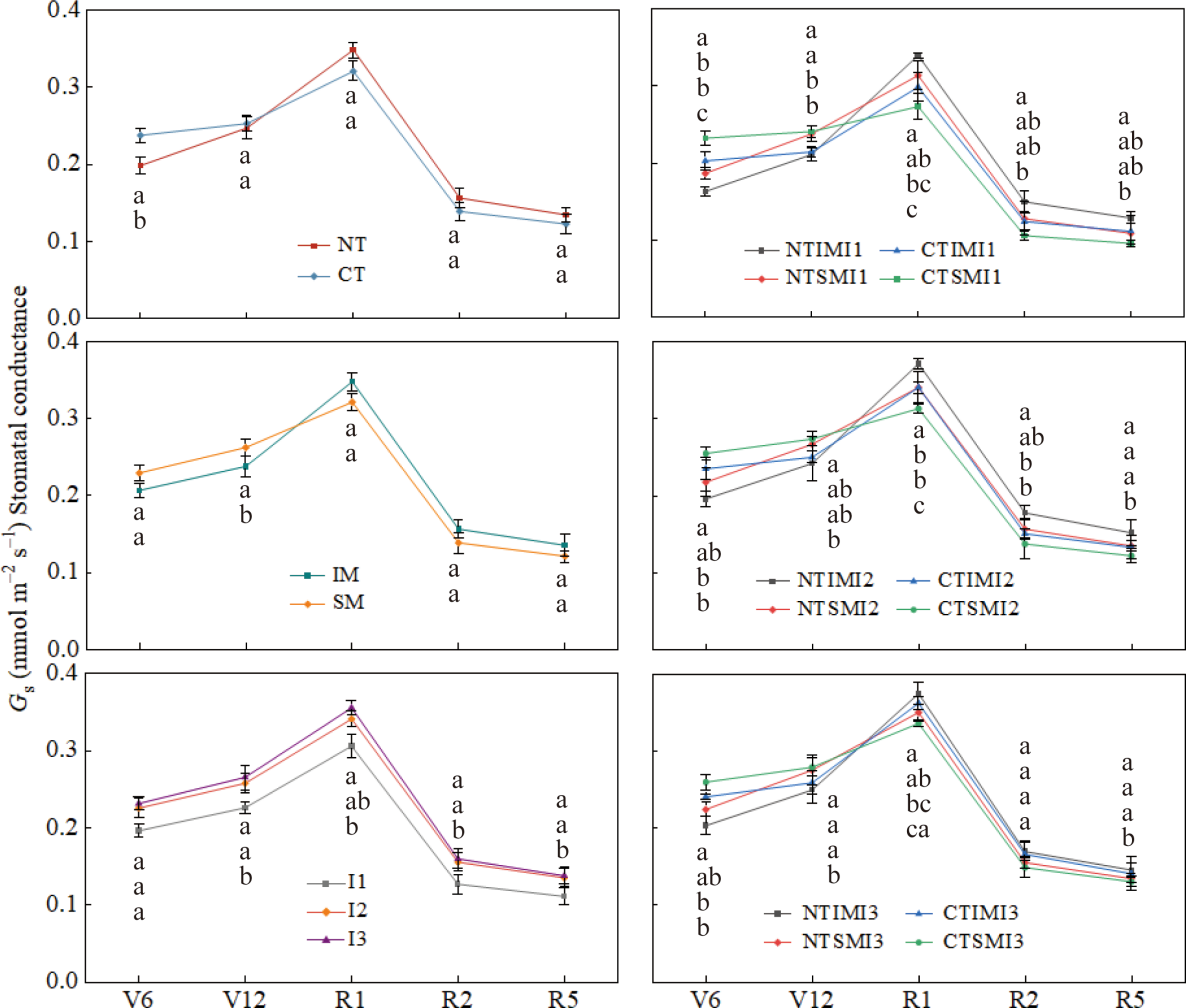
Effect of moderate irrigation under no-tillage on stomatal conductance (Gs) of intercropped maize at different growth stages.

Across treatments, Pn, Gs, and Tr all peaked at silking (**Figures 2**–**4**). Compared with CT, NT slightly reduced Pn, Gs, and Tr at the jointing or early vegetative stage, but increased them by roughly 7–13% from silking to maturity. Similarly, IM showed lower Pn, Gs, and Tr than SM before silking, whereas during the reproductive stage IM exceeded SM by about 6–13%, indicating a delayed but pronounced intercropping advantage. Irrigation had a strong effect: I1 reduced Pn, Gs, and Tr by about 6–21% relative to I3, while I2 generally maintained values comparable to I3. At the treatment-combination level, NT×IM×I2 showed the highest gas-exchange rates from silking to maturity, significantly surpassing CT×IM×I2 and NT×SM×I2, whereas NT×IM×I2 and NT×IM×I3 did not differ significantly and both outperformed NT×IM×I1.

**Figure 5:**
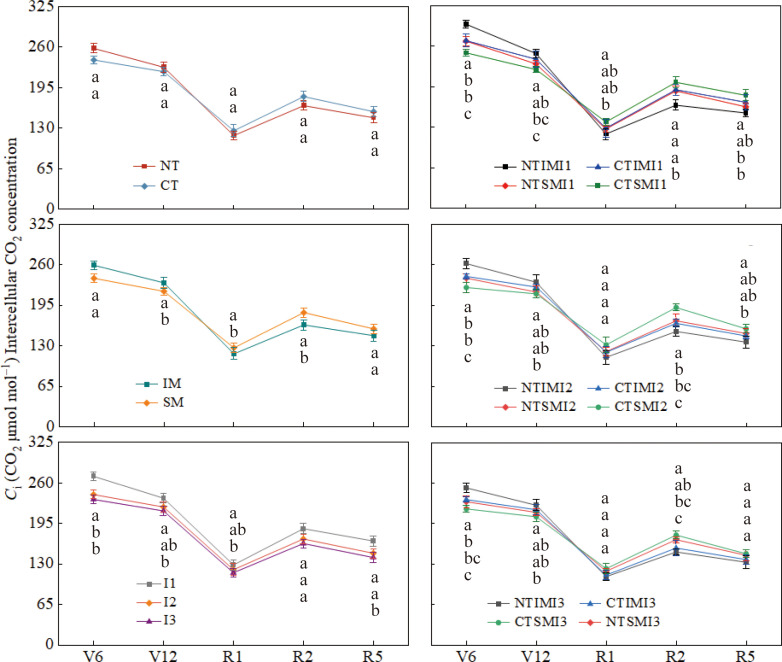
Effect of moderate irrigation under no-tillage on intercellular CO_2_ concentration (Ci) of intercropped maize at different growth stages.

At the jointing stage, NT increased Ci relative to CT, but from silking to maturity it consistently reduced Ci by 6–8%, suggesting improved CO2 assimilation capacity. Similarly, IM showed higher Ci than SM before tasseling, but 7–11% lower values during reproductive stages, indicating enhanced CO2 utilization efficiency under intercropping. Irrigation effects were pronounced during the reproductive period: both I2 and I3 significantly reduced Ci compared with I1, while no difference was observed between I2 and I3 (**Figure 5**).

**Figure 6:**
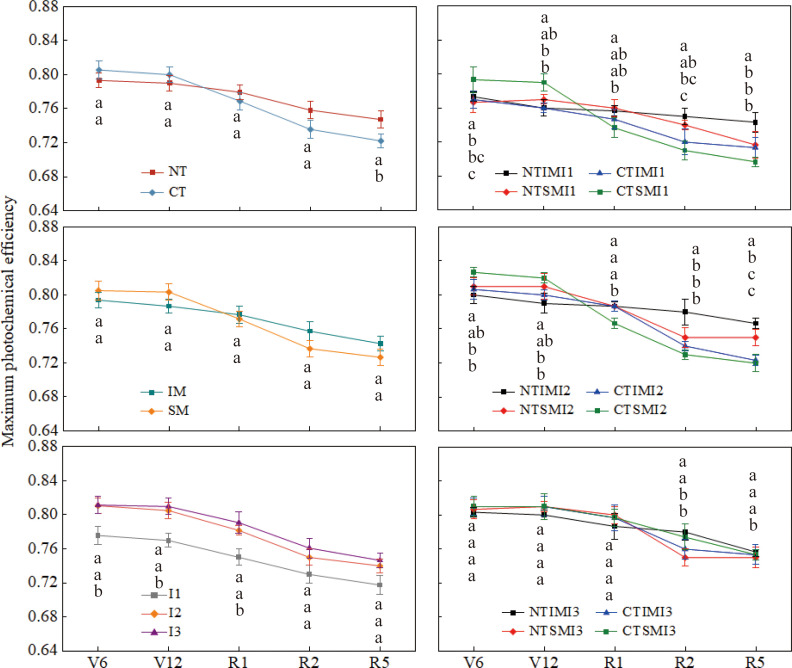
Effect of moderate irrigation under no-tillage on the maximum photochemical efficiency of intercropped maize at different growth stages.

**Figure 7:**
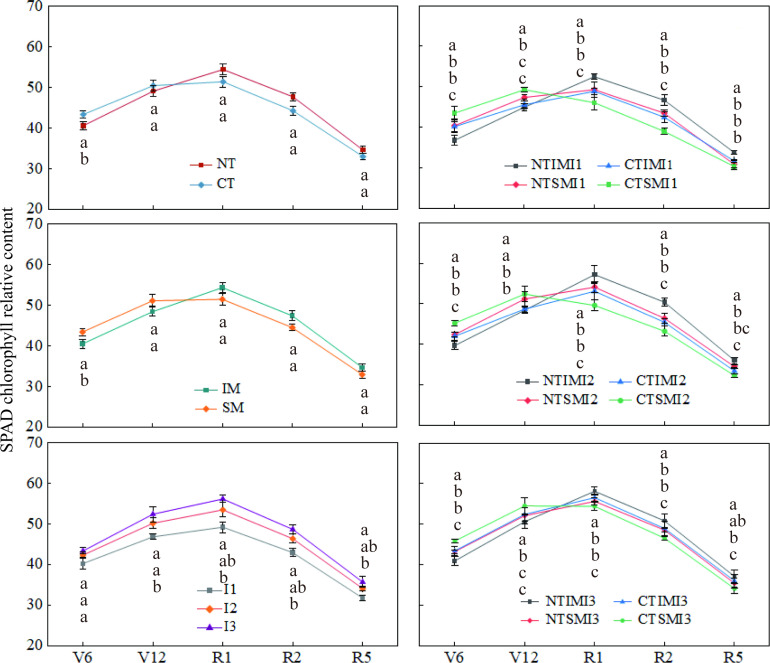
Effect of moderate irrigation under no-tillage on SPAD values of intercropped maize at different growth stages.

Chlorophyll fluorescence and chlorophyll content showed trends consistent with gas-exchange parameters (**Figures 6**, **7**). Compared with CT and SM, NT and IM slightly reduced Fv/Fm and SPAD during early vegetative stages, but increased them by approximately 1–8% from silking to maturity. I1 significantly decreased Fv/Fm and SPAD compared with I2 and I3, whereas I2 and I3 did not differ. At the treatment-combination level, NTIMI2 maintained higher Fv/Fm and SPAD during reproductive stages than CT×IM×I2 and NT×SM×I2, while NTIMI1 consistently showed the lowest values among the NT-based treatments.

### 3.3 Effects of moderate irrigation and no-tillage on the activities of key photosynthetic enzymes in intercropped maize leaves

**Figure 8:**
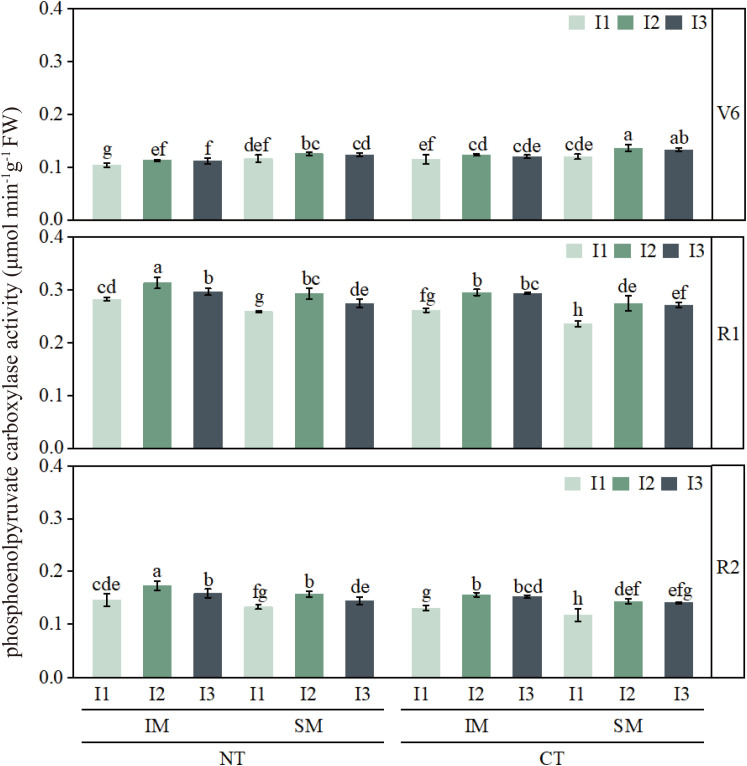
Effect of moderate irrigation under no-tillage on leaf PEPC activity of intercropped maize at different growth stages. Different lowercase letters in the same year mean significant difference at the 0.05 probability level among treatments, and the same applies hereafter.

**Figure 9:**
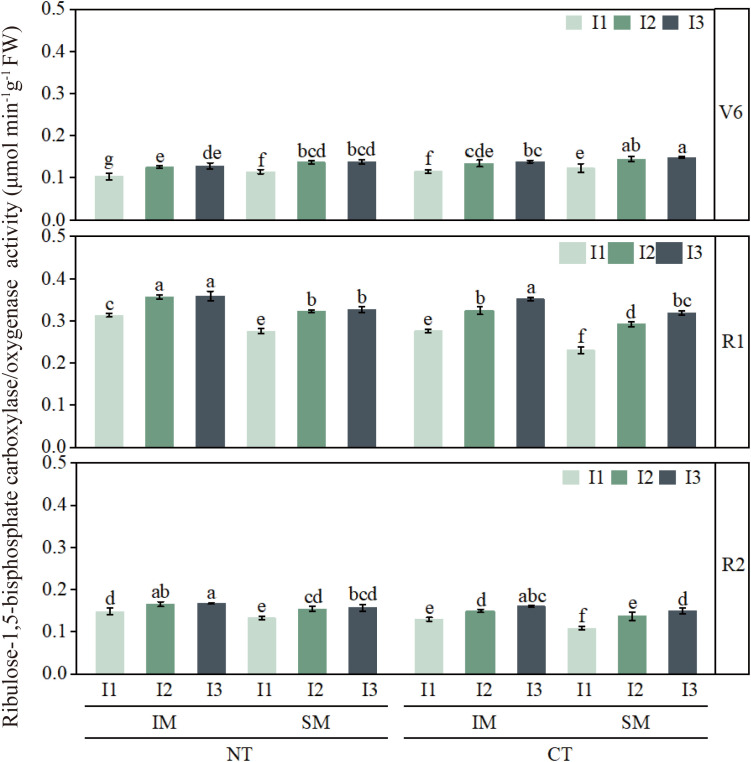
Effect of moderate irrigation under no-tillage on leaf Rubisco activity of intercropped maize at different growth stages. Different lowercase letters in the same year mean significant difference at the 0.05 probability level among treatments, and the same applies hereafter.

**Figure 10:**
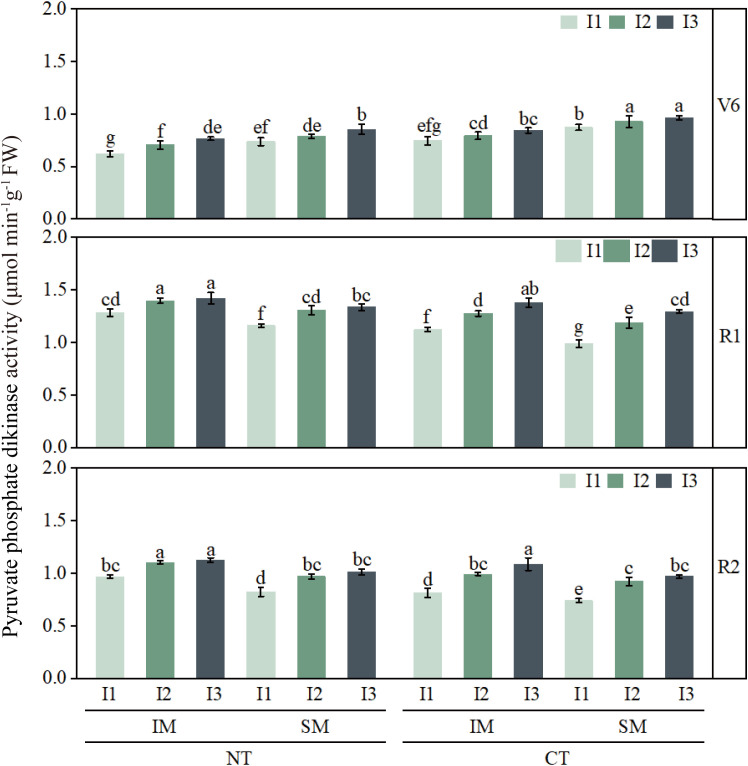
Effect of moderate irrigation under no-tillage on leaf PPDK activity of intercropped maize at different growth stages. Different lowercase letters in the same year mean significant difference at the 0.05 probability level among treatments, and the same applies hereafter.

Activities of PEPC, Rubisco, and PPDK were lowest at the jointing stage and peaked around silking or grain filling (**Figures 8**–**10**). Compared with CT, NT slightly reduced these enzyme activities at jointing, but increased PEPC, Rubisco, and PPDK by approximately 6–13% from silking to grain filling. Similarly, IM showed slightly lower enzyme activities than SM during early growth, but 9–13% higher activities during reproductive stages, indicating a delayed yet clear advantage of no-tillage and intercropping in sustaining photosynthetic capacity.

Irrigation strongly regulated the activities of all three enzymes. Low irrigation (I1) consistently reduced PEPC, Rubisco, and PPDK by about 7–20% compared with high irrigation (I3), whereas moderate irrigation (I2) generally maintained enzyme activities comparable to I3. At the treatment-combination level, NTIMI2 showed the highest enzyme activities from silking to grain filling, exceeding CT×IM×I2 and NT×SM×I2 by roughly 6–14%. In contrast, NT×IM×I1 consistently showed 12–20% lower activities than NT×IM×I2 or NTIMI3, further confirming that moderate irrigation under no-tillage optimizes key photosynthetic enzyme activities in intercropped maize.

### 3.4 Effects of moderate irrigation and no-tillage on the relative expression of key photosynthetic enzyme genes in intercropped maize

**Figure 11:**
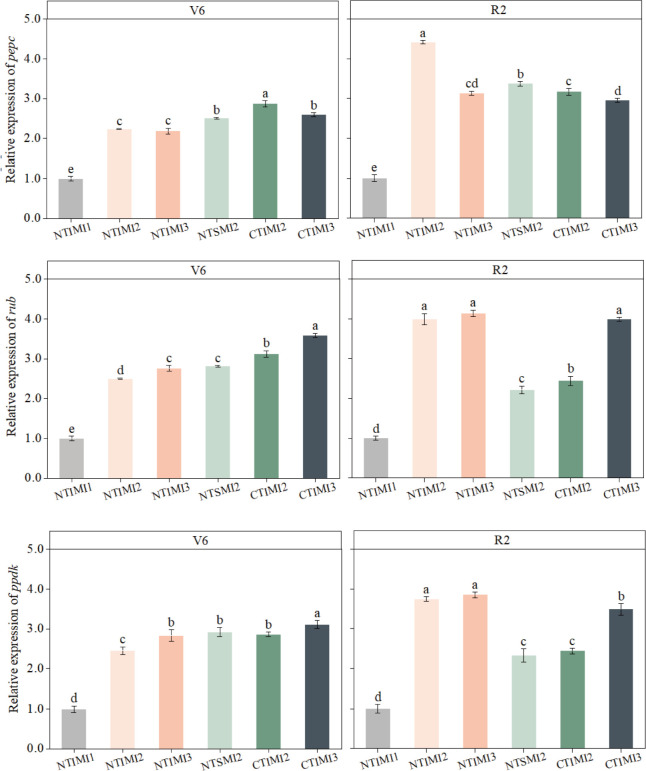
Effect of moderate irrigation under no-tillage on the relative expression levels of *pepc*, *rbcL*, and *ppdk* genes in intercropped maize leaves at different growth stages. Different lowercase letters in the same year mean significant difference at the 0.05 probability level among treatments, and the same applies hereafter.

The relative expression of pepc, rbcL, and ppdk in intercropped maize leaves was generally lower at the jointing stage and markedly higher at the grain-filling stage under NT×IM×I2 compared with conventional tillage or monocropping (**Figure 11**). At jointing, NT×IM×I2 showed slightly lower expression than CT×IM×I2 and NT×SM×I2, whereas at grain filling NT×IM×I2 increased pepc, rbcL, and ppdk expression by approximately 30–80% relative to CT×IM×I2 and NT×SM×I2, indicating a delayed but strong transcriptional advantage of no-tillage and intercropping under moderate irrigation. In contrast, the water-deficit treatment NT×IM×I1 consistently exhibited the lowest transcript levels: from jointing to grain filling, pepc, rbcL, and ppdk expression in NT×IM×I1 was reduced by about 55–75% compared with NT×IM×I2 or NT×IM×I3.

Irrigation regime further modulated these gene-expression responses. Under conventional tillage, CT×IM×I2 did not fully compensate for reduced water supply, and expression of rbcL and ppdk at grain filling remained 13–38% lower than under CT×IM×I3. By contrast, under no-tillage, NT×IM×I2 maintained pepc, rbcL, and ppdk expression at levels comparable to or higher than NT×IM×I3 at grain filling, while strongly exceeding CT×IM×I2 and NT×SM×I2. For all three genes, NT×IM×I1 showed the greatest down-regulation (often >60%) relative to NT×IM×I2 and NT×IM×I3, underscoring that moderate irrigation under no-tillage is critical to sustaining transcription of key photosynthetic enzyme genes in intercropped maize.

### 3.5 Correlation between grain yield and photosynthetic physiological parameters analysis

**Figure 12:**
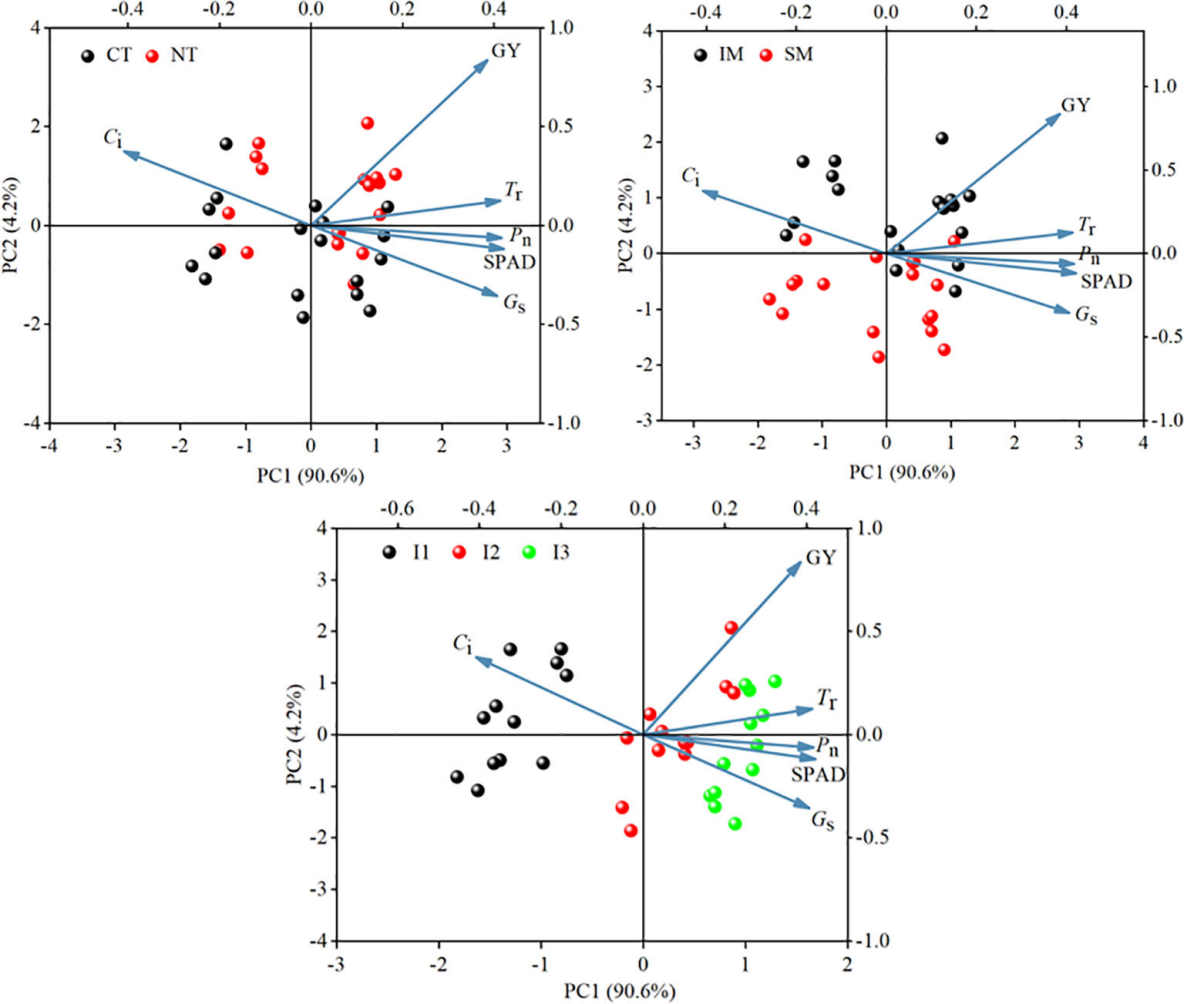
Principal component analysis of grain yield and photosynthetic physiological parameters of intercropped maize under moderate irrigation with no-tillage.

Principal component analysis (PCA) was used to characterize the relationships between grain yield and photosynthetic physiological parameters of intercropped maize under the no-tillage × moderate irrigation condition (**Figure 12**). The first two principal components (PC1 and PC2) explained 94.8% of the total variance, indicating that the major variation in yield–photosynthesis relationships was well captured.

**Figure 13:**
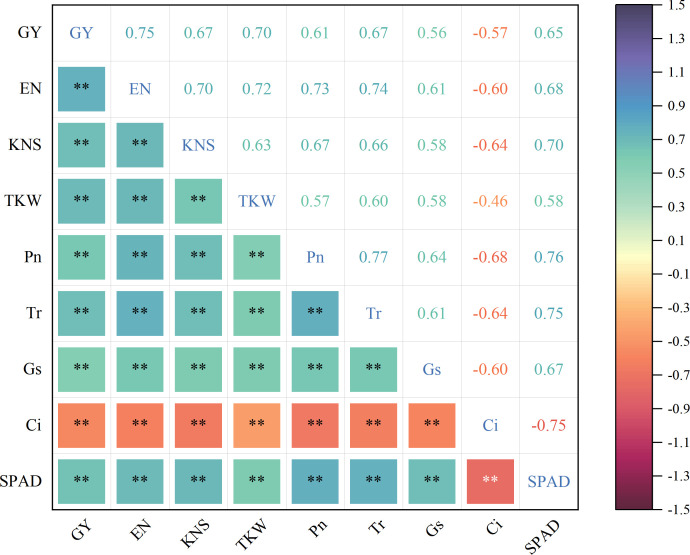
Correlation analysis between grain yield and photosynthetic physiological parameters of maize. **P<0.001.

Correlation analysis among grain yield, yield components, and photosynthetic traits (**Figure 13**) showed that grain yield, ear number, kernel number per ear, and 1000-grain weight were significantly and positively correlated with SPAD, Pn, Gs, and Tr, but significantly and negatively correlated with Ci. These results suggest that maintaining higher leaf greenness and gas-exchange capacity, together with lower intercellular CO2 concentration, is associated with improved yield components and higher grain yield.

## 4 Discussion

### 4.1 Effects of moderate irrigation and no-tillage on grain yield of intercropped maize

In this study, intercropped maize consistently produced a higher grain yield than monocropped maize, but yield stability under water scarcity depended on the concurrent optimization of tillage and irrigation. Notably, integrating no-tillage with moderate irrigation markedly increased intercropped maize yield, achieving a 10.5% higher yield than intercropping under conventional tillage, while maintaining a yield comparable to high irrigation with reduced irrigation input. This indicates that the no-tillage × moderate irrigation combination is an effective management option for water-saving yield stability in oasis maize production.

This yield advantage was associated with sustained source capacity during the critical silking–grain filling period. Under moderate irrigation, no-tillage increased SPAD and peak LAI at silking and slowed their decline during grain filling, suggesting delayed senescence and prolonged photosynthetic duration (Tang and Luo, 2023). These patterns align with previous evidence that intercropping improves canopy light distribution and prolongs the functional period of productive leaves, thereby enhancing resource capture and yield formation (Xu et al., 2020), and that no-tillage can maintain leaf greenness and photosynthetic activity by improving soil water status and reducing late-season decline (Li et al., 2022).

Beyond canopy traits, our results further suggest a biochemical basis for the observed yield stability. No-tillage combined with moderate irrigation enhanced PEPC and Rubisco activities from silking to grain filling, coinciding with higher Pn and Tr, which likely improved CO2 fixation and assimilate supply for kernel growth (Martínez-Goñi et al., 2023). Taken together, the discussion highlights two key mechanisms underlying the water-saving yield stability: maintaining canopy source capacity via delayed senescence and strengthening carbon assimilation capacity via elevated C4-related enzyme activities.

### 4.2 Effects of moderate irrigation and no-tillage on photosynthetic physiological characteristics of intercropped maize leaves

Our results show that leaf photosynthetic performance during the silking–grain filling period was consistently higher in intercropped maize than in monocropped maize, as indicated by higher SPAD and Pn. Moreover, under moderate irrigation, the no-tillage × intercropping combination further increased SPAD, Pn, and Gs, suggesting that this agronomic strategy effectively sustains leaf greenness and stomatal regulation during the late reproductive stage when post-anthesis photosynthesis contributes substantially to assimilate supply and yield formation (Jha et al., 2021). These patterns are broadly consistent with previous reports that cereal–legume intercropping can improve the canopy light environment and nutrient complementarity, thereby delaying senescence and enhancing photosynthetic capacity (Peoples et al., 2009; Yang et al., 2022), and that no-tillage can help maintain post-anthesis photosynthetic potential by improving soil water availability and supporting leaf water status under limited irrigation (Awe et al., 2021). Taken together, our findings indicate that the yield-stabilizing effect of no-tillage with moderate irrigation in intercropping systems is closely linked to maintaining both source activity (SPAD and Pn) and stomatal function (Gs) during grain filling.

Chlorophyll fluorescence responses further support this physiological interpretation. Across irrigation levels, decreasing irrigation reduced Fv/Fm and Y(II), indicating inhibition of PSII photochemical efficiency under stronger water limitation. In contrast, under moderate irrigation, no-tillage maintained higher Fv/Fm and Y(II) during later growth stages, while increasing Y(NPQ) and lowering Y(NO). This combination suggests enhanced regulated energy dissipation and photoprotective capacity, together with reduced risk of photodamage, thereby helping preserve PSII functionality under limited water supply (Li et al., 2018; Zhen and Bugbee, 2020). Similar directions have been reported in studies showing that conservation tillage and intercropping can improve canopy microclimate and soil hydrothermal conditions, which favor the stability of the photosynthetic apparatus under stress (Awe et al., 2021).

In summary, first, moderate irrigation combined with no-tillage in intercropping sustains gas-exchange performance through delayed senescence and improved stomatal regulation; second, it stabilizes PSII photochemistry by strengthening photoprotection and reducing non-regulated energy dissipation. These coordinated responses provide a direct physiological basis for maintaining assimilate accumulation during grain filling and, ultimately, yield stability under water-limited conditions.

### 4.3 Effects of moderate irrigation and no-tillage on the activities of key photosynthetic enzymes and the relative expression of their genes in intercropped maize leaves

In this study, the no-tillage × intercropping system under moderate irrigation showed a clear enhancement of C4-related biochemical capacity during the critical silking–grain filling period. Specifically, compared with low irrigation, moderate irrigation combined with no-tillage increased the activities of PEPC, Rubisco, and PPDK and upregulated the transcription of their corresponding genes, whereas low irrigation consistently resulted in the lowest enzyme activities and gene expression. These results indicate that the yield-stabilizing advantage observed under moderate irrigation is accompanied not only by improved gas exchange but also by strengthened enzymatic and transcriptional support for carbon assimilation.

The temporal pattern is also informative for interpreting the mechanisms. We observed a transient reduction in enzyme activity at jointing under no-tillage intercropping, followed by a pronounced increase from silking to grain filling. This early-stage suppression likely reflects cooler soil conditions and slower early growth under no-tillage with residue and film, while the later-stage enhancement suggests that, as soil temperature constraints diminish, water status becomes the dominant driver of photosynthetic metabolism. Consistent with previous reports, reductions in photosynthetic rate are often associated with declines in key enzyme activities, and maintaining these activities is important for sustaining photosynthetic efficiency and assimilate supply during reproductive growth (Havé et al., 2017; Liu et al., 2018). Our results further support that conservation practices that reduce soil evaporation and improve late-season water availability can help sustain enzymatic capacity and thereby reinforce carbon fixation during grain filling (Awe et al., 2021).

Notably, the contrasting responses between moderate and low irrigation imply a threshold-like behavior: moderate deficit may stimulate enzymatic and transcriptional regulation, whereas stronger water limitation suppresses both enzyme activity and gene expression. This interpretation is consistent with evidence that mild water stress can activate metabolic adjustment and enhance photosynthetic capacity, while severe stress inhibits key processes in the C4 pathway and Calvin cycle (Macedo et al., 2021). Therefore, under moderate irrigation, no-tillage in intercropping systems enhances PEPC, Rubisco, and PPDK activities and the expression of their genes during mid-to-late growth stages, strengthening CO2 carboxylation capacity and supporting higher Pn and related gas-exchange performance. Together, these biochemical and molecular adjustments provide a mechanistic explanation for photosynthetic compensation and yield stability under limited water supply.

## 5 Conclusion

This study demonstrates that, under limited water supply in an oasis irrigation region, coupling no-tillage and maize–pea intercropping with moderate irrigation can trigger a coordinated photosynthesis, enzyme activity and gene expression cascade and achieve less irrigation without yield loss or even yield gains. No-tillage conserved soil water, while intercropping optimized canopy light distribution, jointly delaying leaf senescence and sustaining strong post-anthesis photosynthetic sources. Moderate water stress acted as a mild elicitor, upregulating *pepc*, *ppdk* and *rbcL* expression, enhancing PEPC, PPDK and Rubisco activities, and improving CO_2_ carboxylation capacity and photochemical efficiency. Consistently, this management combination increased Y(II) and Y(NPQ) and reduced Y(NO), maintaining efficient PSII function and limiting non-regulated energy dissipation under water-saving conditions. Beyond maize, the tillage–planting–irrigation triad proposed here provides a transferable framework for designing high-yield, water-efficient, low-carbon C_4_ and cereal–legume systems in arid regions.

## Data Availability

The raw data supporting the conclusions of this article will be made available by the authors, without undue reservation.

## References

[B1] Adil M. Lu S. Yao Z. Zhang C. Lu H. Bashir S. . (2024). No-tillage enhances soil water storage, grain yield and water use efficiency in dryland wheat (Triticum aestivum) and maize (Zea mays) cropping systems: a global meta-analysis. Funct. Plant Biol. 51, FP23267. doi: 10.1071/FP23267. PMID: 38701238

[B2] Awe G. O. Reichert J. M. Holthusen D. Ambus J. V. de Faccio Carvalho P. C. (2021). Characterization of microstructural stability of biochar-amended Planosol under conventional tillage for irrigated lowland rice ecosystem. Soil Tillage Res. 212, 105051. doi: 10.1016/j.still.2021.105051. PMID: 41936479

[B3] Cacefo V. Ribas A. F. Zilliani R. R. Neris D. M. Domingues D. S. Moro A. L. . (2019). Decarboxylation mechanisms of C_4_ photosynthesis in Saccharum spp.: increased PEPCK activity under water-limiting conditions. BMC Plant Biol. 19, 144. doi: 10.1186/s12870-019-1745-7. PMID: 30991938 PMC6469216

[B4] Chen G. Liu M. Zhao X. Bawa G. Liang B. Feng L. . (2024). Improved photosynthetic performance under unilateral weak light conditions in a wide-narrow-row intercropping system is associated with altered sugar transport. J. Exp. Bot. 75, 258–273. doi: 10.1093/jxb/erad370. PMID: 37721809

[B5] Faralli M. Matthews J. Lawson T. (2019). Exploiting natural variation and genetic manipulation of stomatal conductance for crop improvement. Curr. Opin. Plant Biol. 49, 1–7. doi: 10.1016/j.pbi.2019.01.003. PMID: 30851622 PMC6692497

[B6] Ghannoum O. (2009). C_4_ photosynthesis and water stress. Ann. Bot. 103, 635–644. doi: 10.1093/aob/mcn093. PMID: 18552367 PMC2707343

[B7] Guo C. Bao X. Sun H. Chen J. Zhu L. Zhang J. . (2024a). The crucial role of lateral root angle in enhancing drought resilience in cotton. Front. Plant Sci. 15. doi: 10.3389/fpls.2024.1358163. PMID: 38375084 PMC10875062

[B8] Guo C. Bao X. Sun H. Zhu L. Zhang Y. Zhang K. . (2024b). Optimizing root system architecture to improve cotton drought tolerance and minimize yield loss during mild drought stress. Field Crops Res. 308, 109305. doi: 10.1016/j.fcr.2024.109305. PMID: 41936479

[B9] Havé M. Marmagne A. Chardon F. Masclaux-Daubresse C. (2017). Nitrogen remobilization during leaf senescence: lessons from Arabidopsis to crops. J. Exp. Bot. 68, 2513–2529. doi: 10.1093/jxb/erw365. PMID: 27707774

[B10] Jha P. K. Araya A. Stewart Z. P. Faye A. Traore H. Middendorf B. J. . (2021). Projecting potential impact of COVID-19 on major cereal crops in Senegal and Burkina Faso using crop simulation models. Agric. Syst. 190, 103107. doi: 10.1016/j.agsy.2021.103107. PMID: 33623181 PMC7893291

[B11] Jiao F. Ding R. Du T. Kang J. Tong L. Gao J. . (2024). Multi-growth stage regulated deficit irrigation improves maize water productivity in an arid region of China. Agric. Water Manage. 297, 108827. doi: 10.1016/j.agwat.2024.108827. PMID: 41936479

[B12] Khashi U Rahman M. Saati-Santamaría Z. García-Fraile P. (2025). Intercropping of non-leguminous crops improves soil biochemistry and crop productivity: a meta-analysis. New Phytol. 246, 961–971. doi: 10.1111/nph.70037. PMID: 40022473

[B13] Li H. Li L. Liu N. Liu Z. Lu Y. Shao L. (2022). Balanced below- and above-ground growth improved yield and water productivity by cultivar renewal for winter wheat. Front. Plant Sci. 13. doi: 10.3389/fpls.2022.1022023. PMID: 36388545 PMC9659963

[B14] Li Y. Song H. Zhou L. Xu Z. Zhou G. (2018). Tracking chlorophyll fluorescence as an indicator of drought and rewatering across the entire leaf lifespan in a maize field. Agric. Water Manage. 211, 190–201. doi: 10.1016/j.agwat.2018.09.050. PMID: 41936479

[B15] Liu X. Rahman T. Song C. Yang F. Yang W. (2018). Relationships among light distribution, radiation use efficiency and land equivalent ratio in maize-soybean strip intercropping. Field Crops Res. 224, 91–101. doi: 10.1016/j.fcr.2018.05.010. PMID: 41936479

[B16] Macedo V. H. M. Cunha A. M. Q. Cândido E. P. Domingues F. N. da Silva W. L. Lara M. A. S. . (2021). Canopy structural variations affect the relationship between height and light interception in Guinea Grass. Field Crops Res. 271, 108249. doi: 10.1016/j.fcr.2021.108249. PMID: 41936479

[B17] Martínez-Goñi X. S. Miranda-Apodaca J. Pérez-López U. (2023). Could buckwheat and spelt be alternatives to wheat under future environmental conditions? Study of their physiological response to drought. Agric. Water Manage. 278, 108176. doi: 10.1016/j.agwat.2023.108176. PMID: 41936479

[B18] Murchie E. H. Lawson T. (2013). Chlorophyll fluorescence analysis: a guide to good practice and understanding some new applications. J. Exp. Bot. 64, 3983–3998. doi: 10.1093/jxb/ert208. PMID: 23913954

[B19] Pan X. Zhang H. Deng H. Yu S. Zhou C. Li F. (2024). Selecting reasonable soil moisture-maintaining measures to improve the soil physicochemical properties and achieve high yield and quality of purple garlic in the China Hexi Corridor oasis agricultural area. Front. Plant Sci. 15. doi: 10.3389/fpls.2024.1447469. PMID: 39328791 PMC11425794

[B20] Peoples M. B. Brockwell J. Herridge D. F. Rochester I. J. Alves B. J. R. Urquiaga S. . (2009). The contributions of nitrogen-fixing crop legumes to the productivity of agricultural systems. Symbiosis 48, 1–17. doi: 10.1007/BF03179980. PMID: 41933263

[B21] Qu M. Essemine J. Xu J. Ablat G. Perveen S. Wang H. . (2020). Alterations in stomatal response to fluctuating light increase biomass and yield of rice under drought conditions. Plant J. 104, 1334–1347. doi: 10.1111/tpj.15004. PMID: 33015858

[B22] Raza M. A. Yasin H. S. Gul H. Qin R. Mohi Ud Din A. Khalid M. H. B. . (2022). Maize/soybean strip intercropping produces higher crop yields and saves water under semi-arid conditions. Front. Plant Sci. 13. doi: 10.3389/fpls.2022.1006720. PMID: 36407615 PMC9667818

[B23] Rudel T. K. Schneider L. Uriarte M. Turner B. L. I. DeFries R. Lawrence D. . (2009). Agricultural intensification and changes in cultivated areas 1970-2005. Proc. Natl. Acad. Sci. U.S.A. 106, 20675–20680. doi: 10.1073/pnas.0812540106. PMID: 19955435 PMC2791618

[B24] Tang F. Luo H. (2023). Carbon remobilization in the stems of upland cotton as affected by mepiquat chloride and plant density. Field Crops Res. 294, 108864. doi: 10.1016/j.fcr.2023.108864. PMID: 41936479

[B25] Wang L. Peterson R. B. Brutnell T. P. (2011). Regulatory mechanisms underlying C4 photosynthesis. New Phytol. 190, 9–20. doi: 10.1111/j.1469-8137.2011.03649.x. PMID: 21299565

[B26] Xu Z. Li C. Zhang C. Yu Y. van der Werf W. Zhang F. (2020). Intercropping maize and soybean increases efficiency of land and fertilizer nitrogen use: a meta-analysis. Field Crops Res. 246, 107661. doi: 10.1016/j.fcr.2019.107661. PMID: 41936479

[B27] Yang H. Chai Q. Yin W. Hu F. Qin A. Fan Z. . (2022). Yield photosynthesis and leaf anatomy of maize in inter- and mono-cropping systems at varying plant densities. Crop J. 10, 893–903. doi: 10.1016/j.cj.2021.09.010. PMID: 41936479

[B28] Zhen S. Bugbee B. (2020). Far-red photons have equivalent efficiency to traditional photosynthetic photons: Implications for redefining photosynthetically active radiation. Plant Cell Environ. 43, 1259–1272. doi: 10.1111/pce.13730. PMID: 31990071

