# Supplementary material for: Coupling photosynthetic physiology and C4 enzyme regulation enhances grain yield in no-tillage intercropped maize in an irrigated oasis region
Source: Front Plant Sci. 2026 Apr 24;17:1780528. doi: 10.3389/fpls.2026.1780528 (PMC13153119; doi:10.3389/fpls.2026.1780528)
Supplement: Supplementary file 1 [file Table1.docx]

**TABLE S1** Experimental design.

| **Tillage initiatives** | **Planting pattern** | **Irrigation amounts (m^3^ ha^-1^)** | | |
| --- | --- | --- | --- | --- |
|  |  | **I1 (3900)** | **I2 (4650)** | **I3 (5400)** |
| No-tillage (NT) | IM | NT×IM×I1 | NT×IM×I2 | NT×IM×I3 |
|  | SM | NT×SM×I1 | NT×SM×I2 | NT×SM×I3 |
| Conventional tillage (CT) | IM | CT×IM×I1 | CT×IM×I2 | CT×IM×I3 |
|  | SM | CT×SM×I1 | CT×SM×I2 | CT×SM×I3 |

Note: Abbreviations are listed in the Abbreviations section.

**TABLE S2** Irrigation quotas for maize at different growth stages under varying irrigation amounts

| **Irrigation amounts** | **Irrigating water quota (m^3^ ha^-1^)** | | | | | |
| --- | --- | --- | --- | --- | --- | --- |
|  | **Seeding** | **Jointing** | **Big flare** | **Silking** | **Filling** | **Irrigation quota** |
| I1 | 900 | 900 | 900 | 900 | 900 | 4500 |
| I2 | 900 | 1050 | 1050 | 1050 | 900 | 4950 |
| I3 | 900 | 1200 | 1200 | 1200 | 900 | 5400 |

**TABLE S3** The primer sequences for gene expression.

| **Gene Symbol** | **Forward primer(5->3)** | **Reverse primer(5->3)** | **Product length**  **(bp)** | **Tm**  **(℃)** |
| --- | --- | --- | --- | --- |
| LOC542372  *pepc* | TCCACAGTTCGTCTGGTT | GCTTGTCCCTTACATGGC | 108 | 60 |
| LOC542759 *ppdk* | GGACCTTGACGCCATGAA | TAGTGGCTCTCCAGGATGT | 81 | 60 |
| LOC541712  *rbcL* | CTACGACTACATCAGCCAGG | GTCATGAAGTTCTTGGAGAGG | 120 | 60 |
| Maize *actin* | AGATCATGTTCGAGACCTTCA | ACTGGCATACAGAGAGAGCA | 74 | 60 |


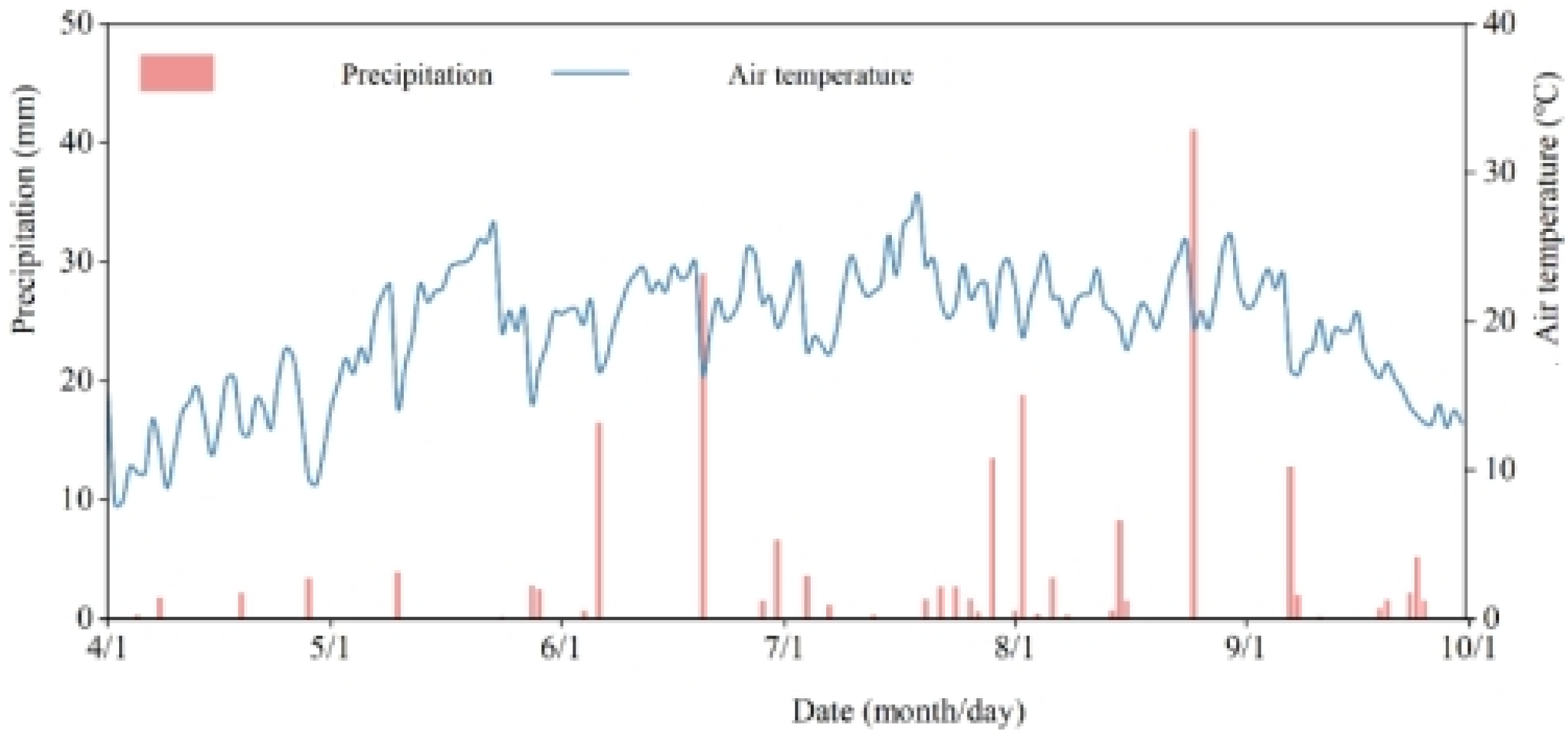


**FIGURE S1** Precipitation and temperature changes in the study area in 2024.
